# Current Status and Issues of Ethical Review for Surgical Research in Japanese University Hospitals

**DOI:** 10.31662/jmaj.2020-0117

**Published:** 2021-12-21

**Authors:** Mariko Doi, Keiko Yukawa, Hajime Sato

**Affiliations:** 1 Department of Health Policy and Technology Assessment, National Institute of Public Health, Wako, Japan; 2 Faculty of Global Nursing, Iryo Sosei University, Kashiwa, Japan

**Keywords:** Surgical research, Research ethics review, Technical review, Study results reporting, Policy guidelines

## Abstract

**Introduction:**

In clinical research, ethical review is required prior to conducting the research. A surgical procedure is a complex intervention with properties that make it more difficult to evaluate rigorously and monitor than drug treatments. This study aimed to clarify the current status and issues in the ethical review and monitoring of surgical research.

**Methods:**

We developed a self-administered questionnaire on surgical ethical review. The questionnaire was distributed to university hospitals in Japan and collected from November 2018 to February 2019. The distributed questionnaire consisted of the reviewed items, items with difficulties, and important items on ethical review. Fisher’s exact test or the chi-square test was used for analysis.

**Results:**

The questionnaires from 39 medical university hospitals were completed with appropriate answers to all items. “Technical review” was conducted at a significantly lower proportion (n = 30/39, 76.9%, p = 0.002). “Evaluation of the progress and results” was also (n = 22/39, 56.4%, p < 0.001). University hospitals in which “technical aspects and ethical review” was regarded the most important and difficult were higher (n = 24/39, 61.5%; n = 26/39, 66.7%, respectively). Respondents considered not only items written in the study protocol but also those on monitoring or oversight of surgical research as difficult.

**Conclusions:**

Our findings suggest that it is necessary to improve the ethical review system and provide supports to conduct an appropriate review for surgical research, e.g., technical aspect review or study progress/result evaluation.

## Introduction

In clinical research that entails work with human participants, ethical review is required prior to starting the research ^(1), (2)^. After several painful lessons in the past decades, various principles or guidelines have been established for human research ^(3), (4), (5), (6)^. Ethical reviews based on these principles are conducted worldwide, and medical researchers conduct clinical studies under these guidelines.

A novel treatment or intervention is expected to improve clinical practice and provide better medical therapeutics, and its ethical aspects are highlighted. Ethical review should be conducted rigorously in implementation research and in managing risks associated with the research. In the case of the developments of new investigational pharmaceutical drugs or medical devices, clinical trials should be reviewed, performed, and overseen by Good Clinical Practice (GCP) guidelines to maintain their scientific, safety, and quality control. In contrast to the ethical review or oversight of a new drug or medical device, norms for surgery have not been established strictly thus far.

A surgical procedure is a complex intervention that is more difficult to evaluate rigorously for safety and efficacy than drug treatments owing to its components ^(7), (8)^. Moreover, surgical research is highly flexible and difficult to monitor and oversee. Previous studies have reported that there is no standardized approach to assist surgeons in assessing ethical challenges in surgical innovation ^(9), (10)^. Gupta et al. proposed few possible oversight frameworks to regulate it, and one was institutional review board (IRB) approval ^(9)^.

Regarding the rules for surgical care and procedures in Japan, the Ministry of Health, Labour and Welfare reissued the Regulation for Enforcement of the Medical Care Act in 2016, and some hospitals were obliged to implement and manage the process of introducing the new medical technology and procedure with high difficulties ^(11)^. The regulation also provided the definition of the novelty and difficulty of medical technology, the skill of the surgeon, the implementation system, and informed consent for hospitals subject to the regulation based on the concept of medical safety. Currently, it is required for hospitals to manage and maintain the surgical care and procedures based on the regulations. A previous study reported the current practice and supervision issues in new clinical surgery ^(12)^. On the other hand, clinical research on surgical research in Japan is covered by the Ethical Guidelines for Medical and Health Research Involving Human Subjects ^(4)^. In this guideline, the ethical review committee should include an expert in natural science, such as a medicine and medical care professional. Medical professionals, however, might not be always specialists in surgery or the research field. The issues of ethical review for surgical research were reported in previous studies, e.g., patients, operators, intervention, comparators, and ethical aspects ^(7), (9), (13)^. Specific problems in surgical research, e.g., randomization, blinding, sham arm, study design, evaluation of scientific validity, and conflict of interest, have remained issues that require attention ^(8), (9), (14), (15)^. However, the actual situation of ethical review and what are considered more difficult are unclear.

Moreover, the Clinical Research Act was implemented on April 2018 in Japan as a result of data falsification in clinical trials by pharmaceutical companies ^(3)^. Although this act is primarily targeted at clinical research within pharmaceutical bodies, Article 2 of this act’s Supplementary Provisions states: “The government considers measures to verify the effectiveness and safety of advanced medical practices that do not necessarily have sufficient scientific knowledge. Based on the results, we will take legal and other necessary measures.” These advanced medical practices include surgery research. Therefore, we hypothesized that there were differences in the implementation and status of ethical review and monitoring of surgical research among university hospitals. The purpose of this study was to clarify the current status and issues in the ethical review and monitoring of surgical research using a questionnaire survey for the condition of university hospitals in Japan.

## Materials and Methods

### Data collection

This study was conducted from November 2018 to February 2019. In this prospective study, data were collected via a self-administered questionnaire ^(15)^ on ethical reviews of research and medical care for surgery by mail via postal services. The questionnaire was distributed to each hospital head of 134 university hospitals in Japan. Respondents received an informative letter regarding our study and a pre-stamped envelope with the address of our department for the respondents’ replies. In addition, an electronic file of the questionnaire was sent to those who wished. The questionnaires were again sent to all non-responding hospitals one month later. All targeted hospitals were confirmed to receive the questionnaire once or twice by phone and asked to answer and send it back.

### Questionnaire

For the purpose of this study, we developed the questionnaire on surgical ethical review. We searched several documents using various databases, including Medline, Web of Science, Social Science Citation Index, and Ichushi for Japanese articles. The search terms included keywords such as ethical examination, surgical technical examination, and new technical examination from the viewpoint of safety and efficacy ^(15)^. We conducted a literature review and summarized previous reports from Japan and other countries ^(7), (8), (9), (10), (13), (14), (16), (17), (18), (19)^. Further, we had a discussion with many domestic and overseas surgeon researchers. Following the discussions with these surgeons, a set of questions was developed for the self-administered questionnaires ^(12)^. The distributed questionnaire consisted of the following chapters: reviewed items to be examined, items with difficulties on ethical review for surgical research, and important items on ethical review. The first chapter consisted of a series of three parts: the necessity of review (one item), the review (six items), and the results (two items) ^(20)^. Chapters 2 and 3 consisted of two similar parts: content related to research implementation and review of study protocol and items on research monitoring or operational management such as management burden (center/research group) or the relationship between research and medical care. Each chapter of this questionnaire consisted of two parts: surgical research and medical care.

### Data analysis

In this study, we analyzed the data on ethical reviews of research for surgery collected using the questionnaire. The answers to the questions were first coded for statistical analysis and double-checked. In the coding of the answer, an answer of “yes” was coded as Yes, and “no” and “no answer” were coded as No. Assuming that the groups for the two items of the questionnaire were independent because each content of the questionnaire was different, Fisher’s exact test or the chi-square test was used. The null hypothesis in those analyses was that no relationship exists on the distribution of two elements that were the responses to the test item and those to the reference; they are independent. The odds ratios obtained from the analysis were interpreted as the degree to the response of “Yes” of the test items compared to those of the references. Statistical analysis was performed using statistical software R version 3.5.2 (R Core Team ^(21)^).

## Results

134 questionnaires were administered. We received a total of 91 respondents. However, some respondents chose not to answer the questionnaire. The questionnaire with all answers blank in a chapter could not be included in the statistical analysis. Meanwhile, 39 questionnaires were completed with valid answers on surgical research in the questionnaire. Thus, the overall response rate to the survey (the number of people who completed the questionnaire to the number of questions answered) was 43%.

### Characteristics of respondents

The characteristics of the questionnaire respondents on surgical research were as follows: Of the university hospitals that responded, 69.2% were national university hospitals, 20.5% were private university hospitals, and 10.3% were public university hospitals. The median number (interquartile range, IQR) of hospital beds of the respondents was 736 (613-856), and the annual median number (IQR) of operations was 7,663 (6,430-9,238). The median number (IQR) of annual approvals for ethical review of clinical studies on surgical research was 3 (0.5-8). Information was also collected on the characteristics of the institutions in which the respondents were based.

### Review items on surgical research

Table 1 shows the review proportion of the items in ethical review for clinical study on surgical research. Review items on the methods and procedures in the study protocol, e.g., ethics, safety, research team system, and informed consent aspects, were examined by almost all medical institutions. Among them, only technical review was conducted at a significantly lower rate at 30 institutions (76.9%, p = 0.002); Fisher’s exact test was used by assuming that the two groups were independent. Regarding the review category related to study progress and results, 35 (89.7%, p = 0.115; compared with the distribution of the “review on ethics”) and 22 (56.4% p < 0.0001; compared with “review on ethics”) medical school hospitals were approached for report or evaluation of the progress and result, respectively. Concerning an item on pre-review other than the contents of the study implementation, 33 (84.6%) medical institutions responded that the judgment on the necessity of review before the ethics review was conducted (p = 0.025; compared with “review on ethics”).

**Table 1.** Review Items in Research Examination on New Surgical Research at Japanese Medical School Hospitals (n = 39).

	Review Items	Yes		No		Fisher’s exact test
		n	(%)	n	(%)	p
Before review	The necessity of review	33	(84.6)	6	(15.4)	0.025
Review for studies	Review on ethics	39	(100.0)	0	(0)	reference
	Review for the efficiency	38	(97.4)	1	(2.6)	ns
	Review on technical aspects	30	(76.9)	9	(23.1)	0.002
	Review on safety	39	(100.0)	0	(0)	ns
	Review on research team systems	39	(100.0)	0	(0)	ns
	Review on informed consent	39	(100.0)	0	(0)	ns
Progress and Results	Report on the progress and results	35	(89.7)	4	(10.3)	0.115
	Evaluation of the progress and results	22	(56.4)	17	(43.6)	<0.001

^*^ Fisher’s exact test was used. ns, difference not existent.

### Items considered important, difficult items

Table 2 shows the proportion of respondents who rated each item as important related to ethical review for clinical study on surgical research. As opposed to items related to the management and oversight of clinical research, the respondents considered that items related to the content should be written in the study protocol as a whole. Specifically, the five items to be included in the protocol involve the novelty, the study implementation conditions, informed consent, and the funding or compensation. Most of all, hospitals in which “technical aspects and ethical review” was regarded the most important were higher (n = 24/39, 61.5%) compared to respondents who considered other items as important (from p < 0.001 to p = 0.023).

**Table 2.** Items That Are Considered Important in Research Ethical Review for New Surgical Research (Up to Three Items to Be Answered) (n = 39).

		Review Items	Yes		No		Chi-square test or Fisher’s exact test		
			n	(%)	n	(%)	OR	95% CI	P-value
Ethical review	Review	Definition of novelty	12	(30.8)	27	(69.2)	0.28	0.11-0.71	0.012
		Implementation conditions	13	(33.3)	26	(66.7)	0.31	0.12-0.79	0.023
		Technical aspects and ethical review	24	(61.5)	15	(38.5)	-	-	reference
		Informed consent	12	(30.8)	27	(69.2)	0.28	0.11-0.71	0.012
		Research expenses and compensation	10	(25.6)	29	(74.4)	0.22	0.08-0.57	0.002
	Release	Study registration and disclosure	1	(2.6)	38	(97.4)	0.02	0.0004-0.12	<0.001
	Results	Evaluation of results	4	(10.3)	35	(89.7)	0.07	0.02-0.27	<0.001
Management and oversight	Effective	Lack of monitoring range, effectiveness, and enforcement	7	(17.9)	32	(82.1)	0.14	0.05-0.39	<0.001
	Burden	Operational effort (research site)	5	(12.8)	34	(87.2)	0.10	0.02-0.32	<0.001
		Operational burden (review committee)	2	(5.1)	37	(94.9)	0.04	0.003-0.17	<0.001
	Discretion	Restrictions of discretion	4	(10.3)	35	(89.7)	0.07	0.02-0.27	<0.001
	Division	Relationship between research and treatment	8	(20.5)	31	(79.5)	0.16	0.06-0.44	<0.001

^*^ Chi-square test or Fisher’s exact test was conducted. OR: Odds ratio

Table 3 shows the proportion of item respondents appraising the difficulties related to ethical review for clinical study on surgical research. Respondents considered not only items written in the study protocol but also those on monitoring or oversight of surgical research as difficult. Concerning items written in the study protocol, most of the respondents in medical schools felt that the most difficult item was on “technical aspects and ethical review” (n = 26/39, 66.7%). Concerning items related to ethical review, the number of medical school hospitals that answered that “informed consent,” “study registration and disclosure,” and “evaluation of results” were difficult was significantly lower (n = 10, 25.6%, p < 0.001; n = 9, 23.1%, p < 0.001; and n = 12, 30.8%, p = 0.003, respectively; compared with the “technical aspects and ethical review”). Regarding items related to operational management and oversight of clinical study, the “lack of monitoring range, effectiveness, and enforcement,” the “operational effort,” and the “restrictions of discretion” were considered difficult.

**Table 3.** Items That Are Difficult to Review in Research on New Surgical Research (Multiple Answers Allowed) (n = 39).

		Review Items	Yes		No		Chi-square test		
			n	(%)	n	(%)	OR	95% CI	P-value
Ethical review	Review	Definition of novelty	20	(51.3)	19	(48.7)	0.53	0.21-1.31	0.250
		Implementation conditions	18	(46.2)	21	(53.8)	0.43	0.17-1.07	0.110
		Technical aspects and ethical review	26	(66.7)	13	(33.3)	-	-	reference
		Informed consent	10	(25.6)	29	(74.4)	0.17	0.06-0.46	<0.001
		Research expenses and compensation	21	(53.8)	18	(46.2)	0.58	0.23-1.46	0.355
	Release	Study registration and disclosure	9	(23.1)	30	(76.9)	0.15	0.06-0.41	<0.001
	Results	Evaluation of results	12	(30.8)	27	(69.2)	0.22	0.09-0.58	0.003
Management and oversight	Effective	Lack of monitoring range, effectiveness, and enforcement	19	(48.7)	20	(51.3)	0.48	0.19-1.19	0.169
	Burden	Operational effort (research site)	18	(46.2)	21	(53.8)	0.43	0.17-1.07	0.110
		Operational burden (review committee)	14	(35.9)	25	(64.1)	0.28	0.11-0.71	0.013
	Discretion	Restrictions of discretion	19	(48.7)	20	(51.3)	0.48	0.19-1.19	0.169
	Division	Relationship between research and treatment	14	(35.9)	25	(64.1)	0.28	0.11-0.71	0.013

^*^ Chi-square test was used.

## Discussion

The present study evaluated the ethical review for surgical research in Japanese university hospitals. Reviewed items on surgical research in the ethical review committee of Japanese university hospitals were unknown. In the present study, we found that aspects on ethics, safety, research team systems, and informed consent for surgical research had been reviewed in most of the ethical review committees. Therefore, we noted that ethics committees of most university hospitals were screened properly. Conversely, some items such as technical aspects or the study progress and results can have a low percentage of review by ethical committees.

In contrast to reviews of ethical aspects or safety and informed consent, approximately 20% of medical institutions were not conducting technical reviews on surgical research. In addition, approximately 61% of medical institutions said that ethical review (technical and ethical) was considered important, and 66% of medical universities face difficulties. The previous study reported the lack of expertise to adequately judge surgical innovation in some IRBs ^(22)^. Moreover, Steiger reported that surgical procedures are usually highly technical and therefore difficult to judge by the IRB ^(23)^. IRBs may have difficulties evaluating surgical innovations either because they lack the necessary expertise or the ability to do so ^(24)^ or because surgical procedures are usually highly complex ^(23)^. In this study, many medical universities might recognize technical review for surgery as important but might find it difficult to implement at the same time because of the absence of an IRB member to understand the details and technique of surgery. Therefore, regarding the technical examination of surgical procedures, support is desired. As described by Steiger ^(23)^, it might be possible to consider relying on external experts for IRB reviews and developing a system for introducing surgical reviews.

In this study, most university hospitals examined items related to clinical trials such as ethics, efficiency, safety, and informed consent. Numerous university hospitals may be focused on reviewing the study plan intensively before a trial begins. Furthermore, in this study, not many medical university hospitals answered that they were conducting examinations on items related to progress and report on results, including adverse events, compared to the other IRB review items. Approximately 90% of medical universities’ hospitals reported receiving the study progress and results, and approximately 60% of them evaluated them. In Japan, the Ethical Guidelines for Medical and Health Research Involving Human Subjects was established in 2014 ^(4)^. This ethics guidelines state that the results, progress, and adverse events of research should be reported to the ethics review committee. Although evaluation of results by the ethical review committee was not specified in the ethical guidelines, evaluation of adverse events was conducted. In Japanese university hospitals, some principal investigators report results to the ethics review committee based on ethical guidelines. However, it was thought that the reporting system based on the ethical guidelines was not operating properly. Reporting of results and adverse events is an important item as required by GCP as well. For early-phase studies, reporting of selective outcomes is likely to lead to overoptimistic assessment of new interventions and under-reporting of adverse effects in many surgical areas ^(25)^. This raises the risk of outcome reporting bias. Therefore, it was considered necessary to take counter responses for medical institutions that had not implemented the reporting of results, including adverse events.

Regarding important items, emphasis is placed on the examination of the research protocol because contents related to research implementation, such as the implementation conditions, informed consent, and definition of novelty, were considered important items. On the other hand, the importance of items, especially the evaluation of the results other than the examination of the study protocol, was not understood, and the implementation ratio was low.

For difficult items, informed consent, trial registration, and evaluation of the results were significantly lower in the scope of the items related to the ethical review. The detail scope of the informed consent and trial registration were stipulated in the ethical guidelines, and current understanding of researchers on trial registration was increasing. It is thought that the understanding of the researchers was improved by the provision of guidelines. On the other hand, the research results have not been evaluated in ethics review committees, and above all, the necessity might not be understood for respondents. They also answered that it was difficult even for items other than those related to conducting research, such as the monitoring and the efforts and burdens involved in operation. It was considered difficult to conduct other monitoring and oversight operations.

Our findings lead to few recommendations for the management of ethical review on surgical research. First, the findings suggest the involvement of technical experts to ensure a technical perspective in ethical reviews. If it is difficult to find an appropriate expert because of a restricted area, we may consider the option of adopting a procedure such as a request of cooperation from the academic society or, if possible, combining it with a review of medical surgery ^(11)^. In a case, it might be useful to provide the guideline written with the detailed procedures. Second, because clinical trial registration also requires reporting of the study results ^(26)^, we would have researchers understand the necessity and awareness of the reporting and evaluation of progress and results. In addition, it is important to design a process for continuous assessment of adverse events, study results, and other events in ethics review. As a result, it might be expected that this will also lead to strengthening of the function of monitoring and/or oversight.

The present study had some limitations. First, this report included a small number of hospitals. Because the questionnaire was distributed to each hospital head, it was also possible that the questionnaire was not passed to the person in charge of clinical research review. Therefore, the findings might not be universally accepted or comprehensive. However, it might be possible that the ethics review committee of the university hospitals that responded in this survey functioned more properly compared to that of university hospitals that did not respond. In that case, it is conceivable that non-responding university hospitals are in a worse condition. Therefore, the results of this study might be underestimated or overestimated. Second, in this study, “no response” of an answer was coded as No in data processing. A missing value was treated as No and included for this analysis. It might be possible to include in “No” a situation in which the problem was not understood, apparent, or recognized as important. The results may be more conservative. However, we paid close attention to this bias and consulted with surgical experts. Third, owing to the space of the questionnaire, the explanation of the question was restricted. It was possible that it may be difficult for the respondents to understand the intent of the question. It might be that some of the non-responders could not answer because they did not understand the questions. When we made phone calls and requested to return the questionnaires, we could sometimes have opportunities to receive the questions and answer them. Lastly, the respondents to this survey were the secretariat or the head of the ethics review committee. A member of an ethics review committee is most likely a physician, but this might differ from the views of researchers, such as physicians conducting clinical research on surgery. It is necessary to understand that this study depended on a situation for committee members.

Further studies are needed to clarify the problems and the useful tips in designing the processes for the involvement of technical experts and the evaluation of research progress and results. It may be useful to conduct an in-depth interview survey for hospitals that were conducting a technical review in this study, those that were evaluating the research results, and those that did not. Useful and detailed information could be obtained for advancing a more appropriate ethical review.

Appropriate evaluation and ethical review in surgical research are difficult to evaluate appropriately because of a complex intervention. In addition, surgical research is highly flexible, and monitoring and oversight are difficult. Our findings show that the ethics, safety, research team system, and informed consent aspects of surgical research were reviewed by most ethics review boards and the technical aspects and the evaluation of research progress and results had a low review proportion. For the improvement of the ethical review for the technical aspects, it is necessary to strengthen the examination system of the ethics review committee. We expected the involvement of experts in the concerned field in ethics review for surgical research. With the involvement of those experts, it is possible to ensure the scientific and ethical aspects in the function of the ethics review committee. Moreover, with the improvement of the evaluation of the study results, that is by consistent reviewing from the beginning of the research to the conclusion, the role of the ethics review committee is expected to further strengthen, including monitoring and oversight. As a result, the quality of clinical research for surgical research is expected to be consistently improved.

## Article Information

### Conflicts of Interest

None

### Sources of Funding

This work was supported by a Health Labour Sciences Research Grant (Practical Research on Medical Technology, Clinical Research Promoting a Research Project) 2018-2019 from the Ministry of Health, Labour and Welfare, Japan [grant numbers H30-Tokubetsu-Shitei-014].

### Acknowledgement

We would like to thank the university hospitals that cooperated with us in this survey.

### Author Contributions

MD: methodology, formal analysis, investigation, data curation, writing - original draft. KY: methodology, validation, writing - review & editing. HS: conceptualization, methodology, writing - review & editing, supervision, project administration, funding acquisition. All authors approved the final version of the manuscript and agreed to be accountable for all aspects of the work in ensuring that questions related to the accuracy or integrity of any part of the work are appropriately investigated and resolved.

### Approval by Institutional Review Board (IRB)

Not required
